# Supplementary material for: Women’s empowerment and experiences of mistreatment during childbirth in facilities in Lucknow, India: results from a cross-sectional study
Source: BMC Pregnancy Childbirth. 2017 Nov 8;17(Suppl 2):335. doi: 10.1186/s12884-017-1501-7 (PMC5688442; doi:10.1186/s12884-017-1501-7)
Supplement: Supplementary file 1 — Mistreatment questions. (DOCX 13 kb) [file 12884_2017_1501_MOESM1_ESM.docx]

Additional file 1. Mistreatment questions

| Did any of the following things happen to you during your most recent delivery? | |
| --- | --- |
| a. Discrimination based on race, ethnicity, or ability to pay | 1.  Yes  0.  No  8.  Don’t know |
| b. Physical abuse (slapping or hitting) | 1.  Yes  0.  No  8.  Don’t know |
| c. Verbal abuse (insult and shouting) | 1.  Yes  0.  No  8.  Don’t know |
| d. Threatening to withhold treatment | 1.  Yes  0.  No  8.  Don’t know |
| e. Lack of information about care provided | 1.  Yes  0.  No  8.  Don’t know |
| f. Ignoring or abandoning patient when in need | 1.  Yes  0.  No  8.  Don’t know |
| g. Delivering alone | 1.  Yes  0.  No  8.  Don’t know |
| h. Denying choice of position during delivery | 1.  Yes  0.  No  8.  Don’t know |
| i. Birth companion(s) not allowed | 1.  Yes  0.  No  8.  Don’t know |
| j. Request or suggestion for informal payments or bribes for better care | 1.  Yes  0.  No  8.  Don’t know |
| k. Unnecessary separation from baby after birth | 1.  Yes  0.  No  8.  Don’t know |
